# Targeting of δ-catenin to postsynaptic sites through interaction with the Shank3 N-terminus

**DOI:** 10.1186/s13229-020-00385-8

**Published:** 2020-10-28

**Authors:** Fatemeh Hassani Nia, Daniel Woike, Victoria Martens, Malte Klüssendorf, Hans-Hinrich Hönck, Sönke Harder, Hans-Jürgen Kreienkamp

**Affiliations:** 1 Institut für Humangenetik, Universitätsklinikum Hamburg-Eppendorf, Martinistrasse 52, 20246 Hamburg, Germany; 2 Institut für Osteologie Und Biomechanik, Zellbiologie seltener Erkrankungen, Universitätsklinikum Hamburg-Eppendorf, Hamburg, Germany; 3 Massenspektrometrische Proteomanalytik, Institut für Klinische Chemie Und Laboratoriumsmedizin, Universitätsklinikum Hamburg-Eppendorf, Hamburg, Germany

## Abstract

**Background:**

Neurodevelopmental disorders such as autism spectrum disorder (ASD) may be caused by alterations in genes encoding proteins that are involved in synapse formation and function. This includes scaffold proteins such as Shank3, and synaptic adhesion proteins such as Neurexins or Neuroligins. An important question is whether the products of individual risk genes cooperate functionally (exemplified in the interaction of Neurexin with Neuroligin isoforms). This might suggest a common pathway in pathogenesis. For the *SHANK3* gene, heterozygous loss of function, as well as missense mutations have been observed in ASD cases. Several missense mutations affect the N-terminal part of Shank3 which contains the highly conserved Shank/ProSAP N-terminal (SPN) and Ankyrin repeat (Ank) domains. The role of these domains and the relevance of these mutations for synaptic function of Shank3 are widely unknown.

**Methods:**

We used purification from a synaptic protein fraction, as well as a variety of biochemical and cell biological approaches to identify proteins which associate with the Shank3 N-terminus at postsynaptic sites.

**Results:**

We report here that δ-catenin, which is encoded by *CTNND2,* an autism candidate gene, directly interacts with the Ank domain of Shank3 at postsynaptic sites through its Armadillo-repeat domain. The interaction is not affected by well-known posttranslational modifications of δ-catenin, i.e. by phosphorylation or palmitoylation. However, an ASD-associated mutation in the SPN domain of Shank3, L68P, significantly increases the interaction of Shank3 with δ-catenin. By analysis of postsynaptic fractions from mice, we show that the lack of SPN-Ank containing, large isoforms of Shank3 results in the loss of postsynaptic δ-catenin. Further, expression of Shank3 variants containing the N-terminal domains in primary cultured neurons significantly increased the presence of coexpressed δ-catenin at postsynaptic sites.

**Limitations:**

Work in model organisms such as mice, and in primary cultured neurons may not reproduce faithfully the situation in human brain neurons. Work in primary cultured neurons was also hampered by lack of a specific antibody for endogenous δ-catenin.

**Conclusions:**

Our data show that the interaction between Shank3 N-terminus and δ-catenin is required for the postsynaptic targeting of δ-catenin. Failure of proper targeting of δ-catenin to postsynaptic sites may contribute to the pathogenesis of autism spectrum disorder.

## Background

Autism spectrum disorder (ASD) is a neurodevelopmental disorder characterized by delayed acquisition of speech, deficits in social interactions and stereotypic behaviours. Molecular genetic studies have shown that the pathogenesis of ASD involves a strong genetic component [1]. Potentially pathogenic mutations were identified in genes coding for synaptic proteins [2]. These include cell adhesion proteins of the Neuroligin and Neurexin families [3]; proteins involved in small G-protein signaling such as Epac or SynGAP [4, 5]; scaffold proteins of excitatory, glutamatergic synapses, including all three members of the Shank family [6–8], and other postsynaptic proteins such as δ-catenin [9]. Based on these findings autism is considered as a synaptic disease or synaptopathy [1], with individual mutations suspected to affect synapse formation and/or synaptic signal transduction and plasticity. Currently it is unclear to what extent the individual products of autism genes interact and work together in common pathways which might affect pathogenesis.

Shank/ProSAP proteins (Shank1-3) are major scaffold proteins of the postsynaptic density (PSD); via multiple interactions they connect different types of glutamate receptor complexes with signalling molecules and the actin cytoskeleton of the dendritic spine [10]. The ability of Shank3 to multimerize via its C-terminal SAM domain has led to the suggestion that formation of Shank clusters is a key event in formation of the large assembly of the postsynaptic density [11]. The human *SHANK3* gene was among the first genes encoding a synaptic protein which was shown to be affected in autism cases. Deletions, frameshift, nonsense and splice site mutations have been observed which lead to loss of function of one *SHANK3* allele. In addition a number of missense mutations have been found in individual autism patients [6, 7]. Interestingly, the relevance of most of these mutations for Shank3 function, and their role in autism pathogenesis is unclear.

Shank proteins consist of multiple protein interaction motifs, including a set of ankyrin (Ank) repeats, SH3, PDZ and SAM domains, as well as a long proline rich region. The Ank repeats are preceded by a ubiquitin like domain at the very N-terminus of Shank1 and Shank3, which we have termed the Shank/ProSAP N-terminal (SPN) domain. The functional relevance of the N-terminal part (SPN and Ank domains) is rather unclear. The SPN domain binds to small G-proteins of the Ras family (e.g. HRas, Rap1) with high affinity, and missense mutations found in ASD patients in this region disrupt G-protein binding [12]. Several interaction partners have been identified for the Ank repeats, including Sharpin, α-fodrin and the hyperpolarization activated cyclic nucleotide gated channel HCN1 [13–15]. In addition, the SPN domain binds to the Ank repeats in an intramolecular manner, thus limiting access to the Ank repeats for its interaction partners [16]. Considering that there is no structural similarity between these interaction partners, it is difficult to define a common interacting motif. In addition, the relevance of these interactions for the function of Shank3 is unclear.

In this study, we aimed to analyse the role of the Shank3 N-terminus in the postsynaptic function of Shank3. We searched for postsynaptic interaction partners of the Shank3 N-terminus; using a biochemical approach, we found δ-catenin as a direct interaction partner of the Ank domain of Shank3 in the PSD. As mentioned above, the *CTNND2* gene coding for δ-catenin is also an autism candidate gene [9]. By overexpression in primary cultured neurons, and analysis of *Shank3* KO mice, we showed that Shank3 directly contributes to the postsynaptic targeting of δ-catenin through this novel interaction mediated by the Shank3 N-terminus.

## Methods

### Expression constructs

Bacterial expression constructs coding for His_6_/SUMO-tagged fusion proteins of rat Shank3 (residues 1-348, SPN-Ank; and 99-348, Ank only) were generated in pET-SUMO (Thermo Scientific) as described [12]. For expression in the human 293T cell line, a construct coding for full-length rat Shank3 with a C-terminal His_6_-Myc-tag was provided by Tobias Böckers (Univ. Ulm, Germany). For several experiments, the *Shank3* cDNA was cloned into pmRFP-N3 (Clontech). Deletion constructs generated by either appropriate restriction sites or PCR amplification of cDNA fragments were also prepared in pmRFP-N3 vectors, leading to expression of Shank3 fragments carrying a C-terminal RFP-tag. A construct coding for N-terminally GFP-tagged full-length rat Shank3 in the pHAGE vector was obtained from Alex Shcheglovitov (Univ. of Utah, Salt Lake City) [12, 17]. Sequence coding for the first 350 amino acids was removed by first introducing a second SalI site at the 3′ end of this part, and then cutting out the SalI fragment. A construct coding for rat Shank1 (Shank1b splice variant) was provided by Carlo Sala (CNR, Milano, Italy). A construct coding for mouse δ-catenin carrying an N-terminal GFP-tag in pEGFP-C1 was obtained from K. Kosik (Univ. of California, Santa Barbara, CA). δ-catenin deletion constructs were generated by PCR based cloning techniques in pEGFP-C1. For expression in neurons, a construct coding for N-terminally RFP-tagged δ-catenin was generated in a modified pSyn vector (gift from Markus Missler; Univ. of Münster, Germany), which is driven by the synapsin promoter. Constructs coding for N-terminally Emerald-tagged α-catenin and Emerald-β-catenin were obtained from Addgene. Plasmids coding for EGFP- and HA-tagged HCN1 were obtained from Roland Bender (UKE, Hamburg, Germany). An expression vector coding for a fusion of EGFP with the C-terminal part of α-Fodrin has been described before [14]. Expression vectors coding for HA-TNIK and HA-TNIK KM (kinase mutant) were obtained from Ken-ichi Kariya (Okinawa, Japan).

### Protein purification

His_6_-SUMO-tagged fusion proteins were expressed in BL21 cells. Proteins were purified from bacterial lysates prepared in native lysis buffer (50 mM NaH_2_PO_4_, 500 mM NaCl, pH 8.0) using Ni–NTA agarose (Qiagen, Hilden, Germany). After elution from beads using 250 mM imidazole, eluted proteins were immediately applied to G-25 columns (GE Healthcare) equilibrated in coupling buffer (0.1 M NaHCO_3_, 0.25 M NaCl). Proteins were eluted in coupling buffer and subjected to coupling to N-Hydroxysuccinimidyl (NHS)-Sepharose at a protein concentration of about 3 mg/ml sepharose beads overnight at 4 °C. Efficiency of purification and coupling steps was verified by SDS-PAGE, followed by Coomassie staining.

### Preparation of the postsynaptic density (PSD) fraction

PSD from mouse brain was prepared according to [18]. Briefly, the forebrains isolated from adult *Shank3* αβ − / − or WT mice were mechanically homogenized in solution A (4 mM Hepes, 0.32 M Saccharose, 1 mM MgCl_2_, 0.5 mM CaCl_2_, pH 7.4, and protease and phosphatase inhibitors cocktails). After several low speed centrifugation steps, a postnuclear supernatant was centrifuged at 13,800 g (4° C, 15 min) to obtain the P2 membrane fraction. This was resuspended in solution B (4 mM Hepes, 0.32 M Saccharose pH 7.4) and applied on a freshly made sucrose gradient consisting of 1.2 M, 1.0 M and 0.85 M sucrose. Ultracentrifugation was performed at 82,500 g for 2 h at 4 °C. The synaptosome fraction was recovered at the 1.0 M/1.2 M interface and resuspended in solution C (containing 12 mM Tris pH 8.1, 0.32 M Saccharose and 1% Triton X-100). After centrifugation at 32,800 g and 4 °C for 20 min, the pellet containing the PSD was further analyzed by solubilization in DOC buffer (50 mM Tris pH 9.0, 1% Na-Deoxycholate, 50 mM NaF, 1 mM Na-Orthovanadate) for further purification of Shank3 interacting proteins. Alternatively, 5 µg/µL of samples were processed for SDS-PAGE and Western Blot by solubilizing in Laemmli sample buffer. The protein concentration was determined using DC™ protein assay (Bio Rad) according to the manufacturer’s instruction. For each condition (coupling to His_6_-SUMO Ank, His_6_-SUMO SPN-Ank or SUMO alone) the isolated PSD fractions from three mice were used.

### Identification of Shank3 binding proteins

Solubilized PSD fraction was incubated with 50 µl of NHS-sepharose beads (coupled to SUMO-Shank3 fragments or SUMO control protein) equilibrated in lysis buffer. Samples were incubated on a rotator for 2 h; after washing (5× in the respective solubilization buffer), bound proteins were eluted with Laemmli sample buffer and applied to SDS-PAGE. Samples were run into first few mm of separating gel, and gel pieces were cut out and processed for mass spectrometric analysis.

### Mass spectrometric analysis

Tryptic in-gel digestion was done according to [19]. Shrinking and swelling was performed with 100% acetonitrile and 100 mM NH_4_HCO_3_. In-gel reduction and alkylation was achieved with 10 mM dithiothreitol, followed by 55 mM iodacetamide (both dissolved in 100 mM NH_4_HCO_3_). Proteins were then digested by covering the gel pieces with a trypsin solution (8 ng/µL sequencing-grade trypsin, dissolved in 50 mM NH_4_HCO_3_ containing 10% actonitrile) and incubating the mixture at 37 °C overnight. Tryptic peptides were extracted with 2% formic acid, 80% acetonitrile. After evaporation, samples were dissolved in 20 µL 0.1% formic acid for LC–MS/MS analysis.

### Protein identification

Analysis of tryptic peptides by LC–MS/MS was achieved by injection of the samples onto a nano-liquid chromatography system (nanoACQUITYy, Waters, Manchester, UK) coupled via ESI to a MS consisting of a quadrupole and an orbitrap mass analyzer (Orbitrap QExcactive, Thermo Scientific, Bremen, Germany). Peptides were separated on a UPLC column, (BEH 130 C18, Waters; 75 μm × 250 mm, 1.7 µm, 100 Ǻ; 200 nL/min) by a linear gradient from 2 to 30% acetonitrile/0.1% formic acid in 120 min. Mass spectra were measured in the positive ion mode. LC–MS/MS analysis was done on MS level over a m/z range from 400–1500.

### Data analysis

With Max Quant (Version 1.5.8.3, [20]), identification was performed with Andromeda against the *Mus* *musculus* SwissProt database (www.uniprot.org). The MaxQuant parameters were set as followed: the precursor mass tolerance was set to 10 ppm, the fragment mass tolerance was set to 0.5 Da and two missed cleavages were allowed for peptide identification; an FDR of 1% was given and a maximum of 5 modifications per peptide were allowed. As a fixed modification the carbamidomethylation on cysteine residues and as variable modifications the oxidation of methionine residues and the acetylation of protein N-terminals were set. The LFQ minimum ratio count was set to 1.

### Cell culture and transient transfection

Human embryonic kidney (HEK) 293T cells were grown in Dulbecco's Modified Eagle Medium supplemented with 10% fetal bovine serum and 1% Penicillin/Streptomycin. Transient transfection of 293T cells was performed using TurboFect Transfection Reagent (Thermo Scientific) according to the manufacturer's instructions.

### Cell lysis and immunoprecipitation

After washing with PBS, cell lysis was performed using immunoprecipitation (IP) buffer (50 mM Tris pH 8, 120 mM NaCl, 0.5% NP40, 1 mM EDTA). Immunoprecipitation was performed using GFP- or RFP-trap beads (Chromotek). Precipitates were washed in IP buffer. Input and precipitate samples were then processed for SDS-PAGE and Western blotting.

### SDS PAGE and Western blot

Proteins were separated on SDS-PAGE under denaturing conditions and transferred to nitrocellulose membrane using a MINI PROTEAN II™ system (Bio-Rad). Membranes were blocked with 5% milk powder/TBS-T and incubated with the indicated primary antibodies overnight at 4 °C followed by HRP-linked secondary antibodies at room temperature for 1 h. Membranes were scanned using a ChemiDoc™ MP Imaging System (Bio-Rad) and images were processed and further analysed using Image Lab Software (Bio-Rad).

### Animals

For preparing primary neuron cultures, brain tissue was isolated from *Rattus* *norvegicus* embryos. Pregnant rats (Envigo; 4–5 months old) were sacrificed on day E18 of pregnancy using CO_2_ anaesthesia, followed by decapitation. Neurons were prepared from all embryos present, regardless of gender (14–16 embryos). To isolate a postsynaptic fraction, the brain tissues of adult (27–39 weeks old) *Shank3* αβ − / − or WT mice were extracted after CO_2_-induced anesthesia and decapitation. *Shank3* αβ-deficient mice used in this study were provided by Tobias Boeckers (Univ. of Ulm, Germany) [21] and have been previously used in our lab [12]. All mouse and rat experiments were approved by, and conducted in accordance with, the guidelines of the Animal Welfare Committee of the University Medical Center Hamburg-Eppendorf (Hamburg, Germany) under permission number Org766.

### Neuron culture and transfection

Primary hippocampal neurons were isolated from E18 rat embryos. The hippocampal tissue was dissected, and hippocampal neurons were extracted by enzymatic digestion with trypsin, followed by mechanical dissociation. Cells were grown in Neurobasal medium supplemented with 2% B27, 1% Glutamax and 1% Penicillin/Streptomycin. Neurons were transfected on DIV7 using the calcium phosphate method.

### Immunocytochemistry

HEK293T cells (one day after transfection) or neurons (DIV14; seven days after transfection) were fixed with 4% paraformaldehyde in PBS for 15 min and permeabilized with 0.1% Triton X-100 in PBS for 5 min at room temperature. After blocking (10% horse serum in PBS) for 1 h at room temperature, cells were incubated with corresponding antibodies overnight followed by 1 h of incubation with Alexa Fluor antibodies. The coverslips were mounted onto glass microscopic slides using ProLong™ Diamond Antifade mounting medium.

### Microscopy

Confocal images were acquired with a Leica Sp5 confocal microscope using a 63 × objective. Quantitative analysis for images was performed using ImageJ.

### Antibodies

The following primary antibodies were used: mouse anti-δ-catenin (BD Trans. Lab; WB: 1:250); mouse anti-GFP (Covance MMS-118P-500, RRID:AB_291290; WB: 1:3000); rat anti-RFP (Chromotek 5F8; WB: 1:1000); Chicken anti-MAP2 (Antibodies-Online ABIN361345, ICC: 1:1000); guinea pig anti-Shank3 (Synaptic Systems # 162 304; WB: 1:1000); mouse anti-PSD-95 (Thermo Fisher MA1-046, RRID:AB_2092361; ICC: 1:500). mouses anti-HA (Sigma Aldrich #H9658; WB: 1:1000). Rabbit anti-cMYC (Sigma #C3956; WB 1:5000).). Mouses anti-α-Tubulin (Abcam #ab7291; WB 1:5000); mouse anti-β-catenin (Cell Signaling; #2698; WB 1:1000); mouse anti-N-cadherin (BD Trans. Lab #610921; WB: 1:3000); mouse anti-NMDAR1 (Merck MAB 363; WB 1:1000); rabbit anti-NMDAR2A (Novus Biologicals NB300-105; WB 1:1000); rabbit anti-NMDAR2B (Novus Biologicals NB300-106; WB 1:1000) HRP-labeled goat secondary antibodies were from Jackson ImmunoResearch and used for WB at 1:2500 dilution. For ICC, Alexa 633 goat anti-mouse IgG (Invitrogen A21050) and Alexa 405 goat anti-chicken IgG (abcam ab175675) were used at 1:1000 dilution.

### Evaluation of data

Statistical significance was performed using Prism8 software (GraphPad, San Diego, CA) and analysed by Student's t-test or one-way ANOVA with post hoc Dunnett's test. All data are presented as mean ± SD.

## Results

### Identification of δ-catenin as an interaction partner of Shank3

**Fig. 1:**
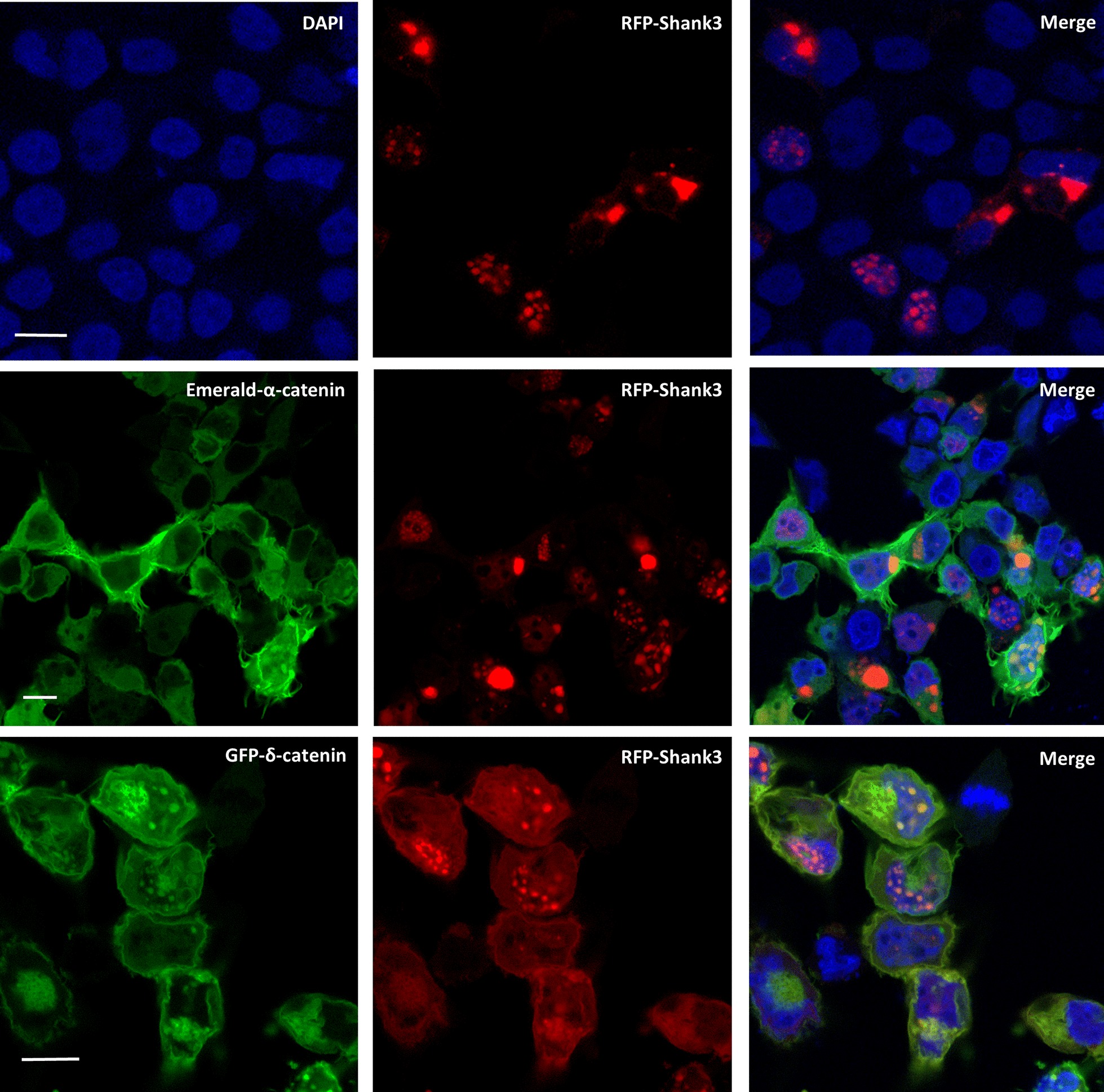
Colocalization of catenin proteins with Shank3 in HEK cells. RFP-tagged Shank3 was expressed either alone (upper panel) or coexpressed with Emerald-tagged α-catenin (middle panel) or GFP-tagged δ-catenin (lower panel) in 293T cells, followed by fixation and confocal microscopic analysis. The results show Shank3 presenting in large nuclear and cytosolic clusters, as described previously [24]; no significant overlap between α-catenin and Shank3; and extensive colocalization of δ-catenin and Shank3 at the plasma membrane (while some δ-catenin/Shank3 clusters remain intracellular). Blue: DAPI staining shows nucleus (scale bar 10 µm)

As the two N-terminal domains of Shank3, i.e. the SPN and Ank domains, were shown by our previous structural work to be closely associated to each other [12], we used a SUMO fusion protein containing both domains as an affinity matrix to purify possible interacting proteins. The same fragment has successfully been used before for crystallization, so we expected it to be properly folded. In addition, we used a fragment containing only the full set of Ank repeats, as well as SUMO alone as negative control, which was purified from cells transformed with the empty vector. We used an isolated PSD fraction as a source for interacting partners in order to specifically target direct postsynaptic interaction partners for the Shank3 N-terminus. For this, PSD was solubilized in Desoxycholate (DOC) containing buffer. A total of four purifications was performed, two with the full N-terminus (SPN-Ank), and two with the Ank repeats only.. Purified proteins were analysed by mass spectrometry, and a list of top candidates was generated by calculating the ratio of signal intensity in Shank3 purified material, divided by material purified on the SUMO matrix (Table 1). This list contained in all four purifications, for both SPN-Ank and Ank-only purified material, several proteins of the catenin family, including α-, β- and δ-catenins. As different catenin proteins interact with each other directly, and also indirectly via their association with cadherins [22], it was initially unclear whether this was due to direct binding of Shank3 to any of these proteins. In addition, we have recently reported that the central PDZ domain of Shank3 binds to β-catenin [23]. It was therefore important to identify which catenin variant binds directly to the Shank3 N-terminal domains. For this, RFP-tagged Shank3 was expressed in 293T cells either alone or together with fluorescently tagged versions of catenins (Fig. 1). As observed previously [23, 24], Shank3 forms large clusters in these cells, which were found partly in cytosolic structures but also to a larger extent in small nuclear clusters. Coexpression with α-catenin did not change this pattern, and we observed little colocalization of α-catenin with Shank3 as α-catenin was found mainly in diffuse cytosolic, as well as membrane attached structures. We have described before that β-catenin, in agreement with its known nuclear role, was found mostly in nuclear clusters where it was almost perfectly colocalized with Shank3 [23]. Finally, coexpression with δ-catenin led to a relocalization of Shank3 to a plasma membrane associated pattern, which was not seen in the absence of δ-catenin. δ-catenin and Shank3 were colocalized here, in agreement with the membrane attachment of δ-catenin by palmitoylation. In summary, δ-catenin was able to change the subcellular location of Shank3, whereas α-catenin did not affect the localization of Shank3.

**Table 1:** Result of mass spectrometric analysis of Shank3 N-terminus postsynaptic interaction partners

Protein names	Gene names
Armadillo repeat protein deleted in velo-cardio-facial syndrome	*Arvcf*
14-3-3 protein sigma	*Sfn*
Catenin alpha-1	*Ctnna1*
Versican core protein	*Vcan*
Probable ubiquitin carboxyl-terminal hydrolase FAF-X	*Usp9x*
Arf-GAP with GTPase, ANK repeat and PH domain-containing protein 3	*Agap3*
Pancreatic alpha-amylase	*Amy2*
SH3 and multiple ankyrin repeat domains protein 3	*Shank3*
Catenin delta-2	*Ctnnd2*
Dynactin subunit 1	*Dctn1*
Myosin phosphatase Rho-interacting protein	*Mprip*
Cadherin-2	*Cdh2*
F-box only protein 41	*Fbxo41*
Rho GTPase-activating protein 39	*Arhgap39*
Disks large-associated protein 4	*Dlgap4*
Synaptosomal-associated protein 47	*Snap47*
Claudin-11	*Cldn11*
Ras/Rap GTPase-activating protein SynGAP	*Syngap1*
Voltage-dependent N-type calcium channel subunit alpha-1B	*Cacna1b*
Arf-GAP with GTPase, ANK repeat and PH domain-containing protein 2	*Agap2*
Disks large homolog 3	*Dlg3*
SRC kinase signaling inhibitor 1	*Srcin1*
Catenin beta-1	*Ctnnb1*
Disks large homolog 4	*Dlg4*
Voltage-dependent R-type calcium channel subunit alpha-1E	*Cacna1e*
Catenin delta-1	*Ctnnd1*
Latrophilin-1	*Lphn1*
Sorbin and SH3 domain-containing protein 1	*Sorbs1*
Brain-specific angiogenesis inhibitor 2	*Bai2*

The isolated postsynaptic fraction from mouse brain was subjected to affinity purification with either His_6_-SUMO/Shank3 N-terminal fusion proteins or His_6_-SUMO as negative control. Both sets of samples were analysed by mass spectrometry. The label free quantification (LFQ) intensity signals obtained from the negative control samples were divided by the LFQ intensity signals obtained from the Shank3 N-terminus purified samples. Partners with the highest residual LFQ intensity signals are listed. Proteins of the catenin and cadherin families are highlighted in yellow. A similar result was obtained in four separate purifications, two with the complete N-terminus (shown here) and two with the Ank repeats of Shank3 only

### Mapping of interacting domains

**Fig. 2:**
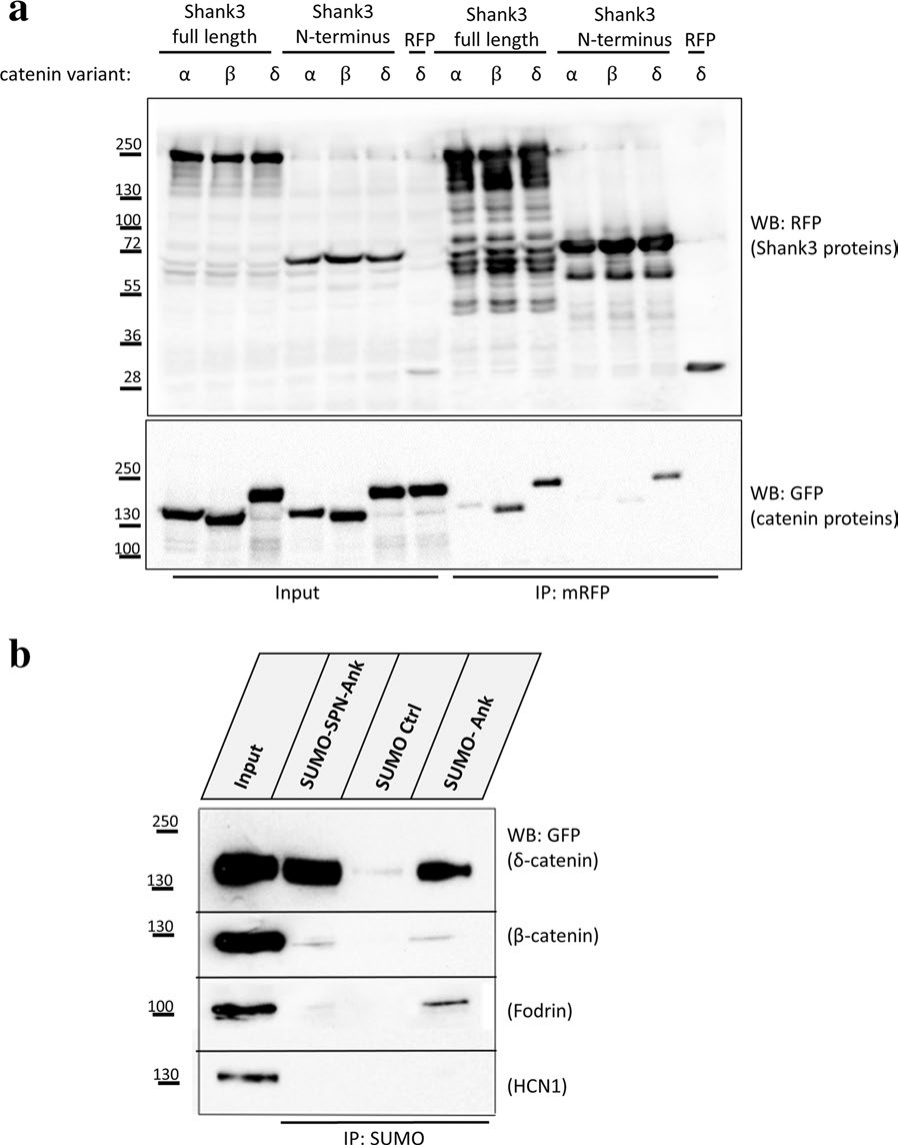
**a** Interaction of catenin proteins with Shank3. 293T cells coexpressing mRFP-Shank3 with GFP-/Emerald-tagged catenin variants were lysed and subjected to immunoprecipitation using RFP-trap. Input and precipitate samples were analysed by Western blotting. The result shows that β- and δ-catenin (but not α-catenin) bind to full length Shank3, while only δ-catenin binds to the Shank3 N-terminal fragment (SPN-Ank). **b** 293T cells expressing GFP-tagged versions of the proteins indicated were lysed (Input samples) and subjected to a pulldown assay using immobilized His_6_-SUMO fusion proteins of the complete Shank3 N-terminus (SPN-Ank), the SUMO control protein or the Ank repeats alone. δ-catenin shows strong, specific binding to the Shank3 N-terminus when compared to the other interaction partners; Fodrin binds to the isolated Ank domain only, whereas HCN1 does not show any binding neither to Ank nor to the SPN-Ank fragment

Verification of the initial mass spectrometric results in 293T cells showed a robust coimmunoprecipitation of δ-catenin but not of α-catenin upon coexpression with full length Shank3 (Fig. 2a). Therefore we focused further on δ-catenin as mutations in the coding *CTNND2* gene have been observed in a few autism cases [9]. Previous work from our group had indicated that δ-catenin is in a complex with Shank3, and this was supported by in vivo data from Shank3-overexpressing mice [24, 25]. However, it was unclear whether there is a direct contact, and which domains of the interacting proteins are involved.

Here we used a set of deletion constructs of *Shank3* to further confirm the site of interaction. These experiments clearly show that the N-terminal part (SPN-Ank domains) is sufficient for binding to δ-catenin (Fig. 2a). Furthermore we confirmed the results of our colocalization study, as we observed that both β- and δ-catenin but not α-catenin bind to Shank3, and that both catenins use different binding sites on Shank3: δ-catenin binds to the N-terminal domains whereas β-catenin binds only to full-length Shank3 (Figs. 2a and Additional file 1: Fig. S1). This is in agreement with recent work where we showed that Shank3 directly binds to β-catenin via its PDZ domain [23].

To further delineate the relevance of the two N-terminal domains (SPN and Ank), we performed a pulldown experiment using the bacterially expressed, SUMO-tagged fragments of Shank3 (Fig. 2b). Here, two additional, known interaction partners of the Shank3 N-terminus were included, namely α-fodrin [16, 26] and HCN1 [15]. In this experiment we could clearly distinguish different modes of binding to the Shank3 N-terminus, as δ-catenin bound to both short (only Ank) and the longer fragment of Shank3 (SPN-Ank); and α-fodrin efficiently interacted only with the fragment of Shank3 that lacks the SPN domain. This is in agreement with previous data from our lab, where we showed that the intramolecular interaction between SPN and Ank domains prevents access of α-fodrin to the Ank repeats [16]. No binding was observed for β-catenin which was used as a negative control here. Surprisingly, no binding was detected also for HCN1 which has been previously reported as a direct interaction partner of the Shank3 Ank domain (also see Additional file 1: Fig. S2).

**Fig. 3:**
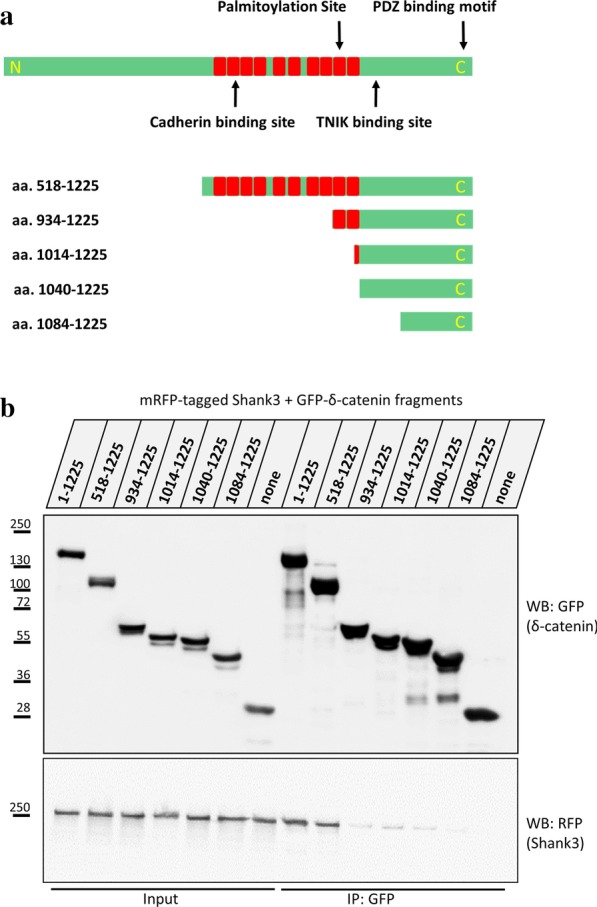
Mapping the Shank3 binding site on δ-catenin. **a** The domain structure of δ-catenin, and the truncated proteins used in this study. The Armadillo repeats are indicated in red and the binding sites for N-cadherin and PDZ ligands as well as the phosphorylation and palmitoylation sites are indicated by arrows. **b** GFP-tagged fragments of δ-catenin were coexpressed with RFP-tagged full-length Shank3. Cells were lysed and GFP-tagged proteins were immunoprecipitated using the GFP-trap matrix. Input and IP samples were analysed by Western blotting. The results show that the Arm repeats of δ-catenin bind to Shank3

Using deletion constructs that produce δ-catenin fragments lacking different domains such as the N-terminus, the Armadillo (Arm) repeat region or the portions of C-terminus, we mapped the Shank3 binding site on δ-catenin (Fig. 3a). The result of coimmunoprecipitation of these truncated fragments of δ-catenin with Shank3 showed that removing the Arm repeat region strongly diminished the interaction with Shank3, indicating that the Arm repeat region of δ-catenin is an interaction motif for the Shank3 Ank repeats (Fig. 3b).

### Effect of *SHANK3* missense mutations

**Fig. 4:**
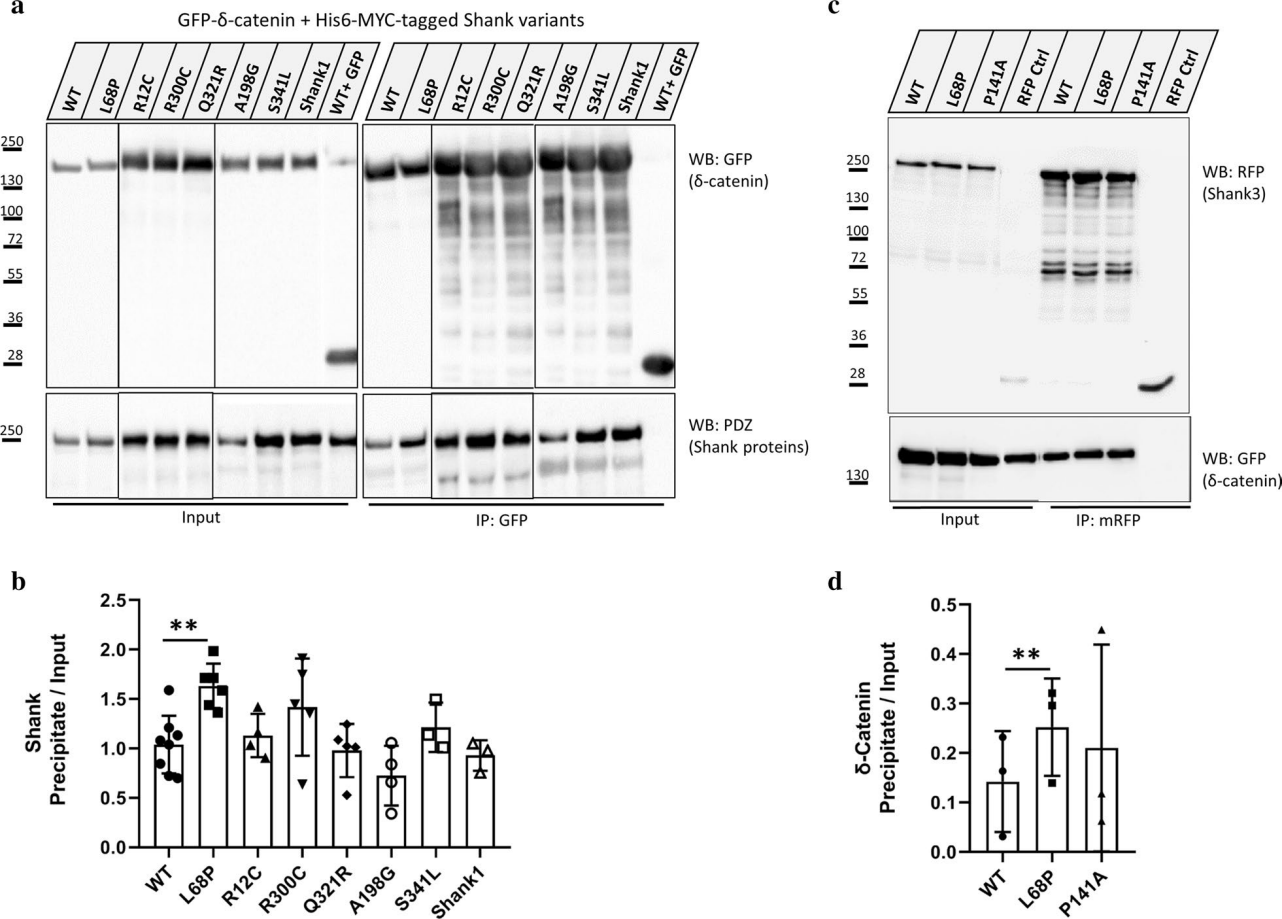
Effect of Shank3 N-terminal variants on binding to δ-catenin. **a** Shank3 WT and variants carrying ASD mutations, as well as Shank1, were coexpressed in 293 cells with GFP-tagged δ-catenin. Cell lysates were subjected to coimmunoprecipitation of GFP-δ-catenin using GFP-trap. **b** Quantification of the results in A, shown. as the ratio of Shank3 IP/input signals. The L68P mutation significantly increases the binding of δ-catenin to Shank3. Shank1 is also able to interact with δ-catenin via its N-terminal domain (*n* = 4, one-way ANOVA with Dunnett’s Test, **p < 0.01, mean ± SD). **c** δ-catenin was coexpressed with RFP-tagged Shank3 WT or variants carrying L68P and P141A mutations; cells were lysed and lysates were subjected to immunoprecipitation using RFP-trap. **d** Quantification of the results in **c**, shown as the ratio of GFP-δ-catenin IP/input signals. While the effect of L68P is again significant in improving binding of δ-catenin to Shank3, the de novo mutation P141A slightly (but non-significantly) improves the binding (*n* = 3, one-way ANOVA with Dunnett’s Test, **p < 0.01, mean ± SD)

So far, seven missense mutations associated with ASD have been identified in the Shank3 N-terminus [6, 7, 27–29]. We analysed, by coexpression and coimmunoprecipitation whether these mutations interfere with the ability of Shank3 to bind to δ-catenin (Fig. 4a, c). Here only the L68P mutation in the SPN domain showed a significant impact as it increased binding to δ-catenin (Fig. 4b, d). Leucine 68 is located in the hydrophobic core of the SPN, and this mutation is likely to unfold this part of the protein [12, 16]. As a consequence, interaction partners of the Ank repeats can access this domain better, as was shown previously for Sharpin and α-fodrin [16]. Thus the increase in δ-catenin binding may be explained by this mechanism. However, in previous experiments we observed a much stronger increase for α-fodrin binding induced by the L68P mutation, indicating that δ-catenin binds to the Ank repeats of Shank3 in a different manner than α-fodrin [16]. In this experiment, we also included Shank1, which has an SPN domain and a very similar set of Ank repeats when compared to Shank3 (Fig. 4a). A strong interaction of δ-catenin was also observed with Shank1, suggesting a similar mode of binding for δ-catenin to Shank1 and Shank3 (Fig. 4b).

### Effect of posttranslational modifications of δ-catenin

We further investigated the effect of posttranslational modifications of δ-catenin on its binding to Shank3. δ-catenin is a known neuronal substrate of the TRAF2 and NCK-interacting protein kinase (TNIK) with a known TNIK phosphorylation site at Thr1064 [30]. To investigate the effect of TNIK phosphorylation on the interaction between Shank3 and δ-catenin, both proteins were expressed in 293T cells together with either an HA-tagged WT TNIK or a TNIK "kinase dead" protein. The results of coimmunoprecipitation showed that the presence of active WT TNIK, and therefore phosphorylation of δ-catenin under this condition does not change the state of interaction between Shank3 and δ-catenin (Additional file 1: Fig. S3A). Similarly, mutation of Thr1064, one of the main phosphorylation sites, to Ala or to Glu (as a phosphor-mimic) did not affect binding of δ-catenin to Shank3 (Additional file 1: Fig. S3B). Furthermore, δ-catenin is known to be palmitoylated by the palmitoyl transferase DHHC5 at two adjacent cysteine residues (Cys960 and Cys961) within the Arm repeats [31]. We mutated these two residues to serine; again, coexpression/coimmunoprecipitation experiments showed that this modification does not lead to an altered interaction with Shank3 (Additional file 1: Fig. S3B).

### Targeting of δ-catenin to postsynaptic sites by Shank3

**Fig. 5:**
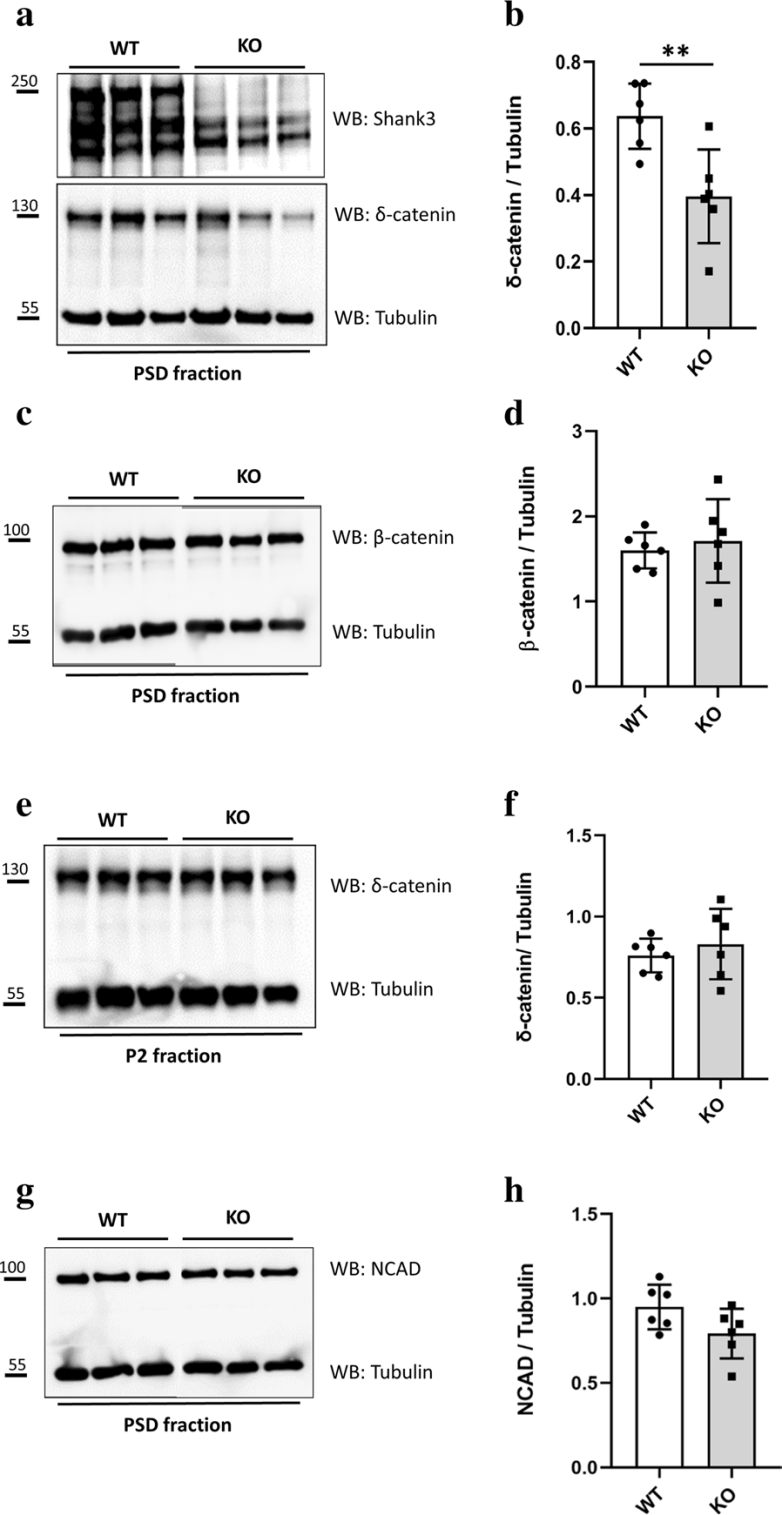
The level of catenin proteins in the postsynaptic fraction of *Shank3* KO mice. **a** The PSD fractions from WT and KO mice lacking the larger SPN-Ank-containing isoforms of Shank3 were prepared and analyzed by Western blotting using the antibodies indicated. Three major isoforms of Shank3 are present, according to [21]. The larger bands of Shank3 (containing α, β variants) detected in the WT PSD samples are absent in the PSD of *Shank3* KO mice, indicating the lack of isoforms of Shank3 containing N-terminal SPN-Ank domains. **b** The level of δ-catenin quantified as the δ-catenin/Tubulin ratio shows a significant decrease in the PSD fraction of the *Shank3* KO mice. **c, d** The representative blot and graph show that the postsynaptic localization of β-catenin does not change in the absence of the Shank3 N-terminus. **e, f** The representative blot and graph show the level of δ-catenin in the P2 fraction of *Shank3* KO animals containing membrane associated proteins is not affected by the absence of Shank3 N-terminus. **g, h** Using a specific antibody for detecting N-cadherin in the isolated PSD fractions of *Shank3* KO and WT mice shows that the level of N-cadherin is slightly but not significantly reduced in the postsynaptic fraction KO mice lacking the larger SPN-Ank-containing isoforms of Shank3 (*n* = 6 WT and 6 KO animals for all graphs, **p < 0.01, unpaired *T*-test; data are shown as mean ± SD)

To determine the in vivo relevance of the interaction between Shank3 and δ-catenin, we compared the enrichment of δ-catenin in an isolated PSD fraction of WT versus *Shank3* KO mice. PSD was prepared using several centrifugation steps, including ultracentrifugation on a sucrose density gradient, followed by extraction with mild detergent (Triton X-100). This is an established method, which produces 10–20 fold enrichment of core postsynaptic scaffold proteins such as Shank or PSD-95, as well as NMDA receptors. Tubulin, which is not believed to be postsynaptic, is however still present as a known contaminant and can be used for normalizations (see Additional file 1: Figure S4). The *Shank3* KO mice used in this study lack the large α and β isoforms of Shank3 containing the SPN, Ank and SH3 domains, whereas other forms of Shank3 initiating at the PDZ domain are still present (Fig. 5a) [21]. The PSD fractions of six animals per each condition were tested by detecting δ-catenin with a specific antibody; signals were normalized by signals obtained with an anti-tubulin antibody (Fig. 5a). Here, we observed that the level of postsynaptic δ-catenin is significantly reduced in the absence of the large Shank3 isoforms carrying the site of interaction for δ-catenin (Fig. 5b). In contrast, the postsynaptic level of β-catenin is not altered in the postsynaptic density of these *Shank3* αβ deficient mice. This is in agreement with our previous work [23], as interaction of β-catenin with Shank3 is not dependent on the N-terminus of Shank3, but requires the PDZ domain (Fig. 5c, d). By comparing the P2 membrane fractions of WT and *Shank3* KO mice (Fig. 5e), we observed that the level of membrane associated δ-catenin is not different between *Shank3* WT and KO animals (Fig. 5f). Thus, the overall level of δ-catenin is not affected by loss of the interaction with Shank3. It should be noted here that, though δ-catenin can be readily detected in the PSD fraction, it does not appear to be particularly enriched when comparing with the P2 fraction. Thus, δ-catenin is not a core, exclusive component of the PSD such as known postsynaptic scaffolds Shank or PSD-95. Instead, the δ-catenin concentration at postsynaptic sites appears to be regulated by other factors, and our data indicate that Shank3 is involved in the postsynaptic trafficking or the stabilization of δ-catenin at the synapse.

It has been reported that the absence of δ-catenin leads to a reduced level of N-cadherin (NCAD) at postsynaptic sites [32]. Therefore, to analyse whether Shank3, through regulating the level of δ-catenin in the postsynaptic density, also contributes to targeting of N-cadherin to the postsynaptic sites, we compared the level of NCAD in the postsynaptic density of the same WT and *Shank3* KO animals mentioned above. Here, the level of postsynaptic N-cadherin was slightly, but not significantly reduced in the absence of the long, δ-catenin binding Shank3 isoforms. This may indicate a compensatory effect of other catenins still present at postsynaptic sites in the absence of specific Shank3 isoforms (Fig. 5g, h).

**Fig. 6:**
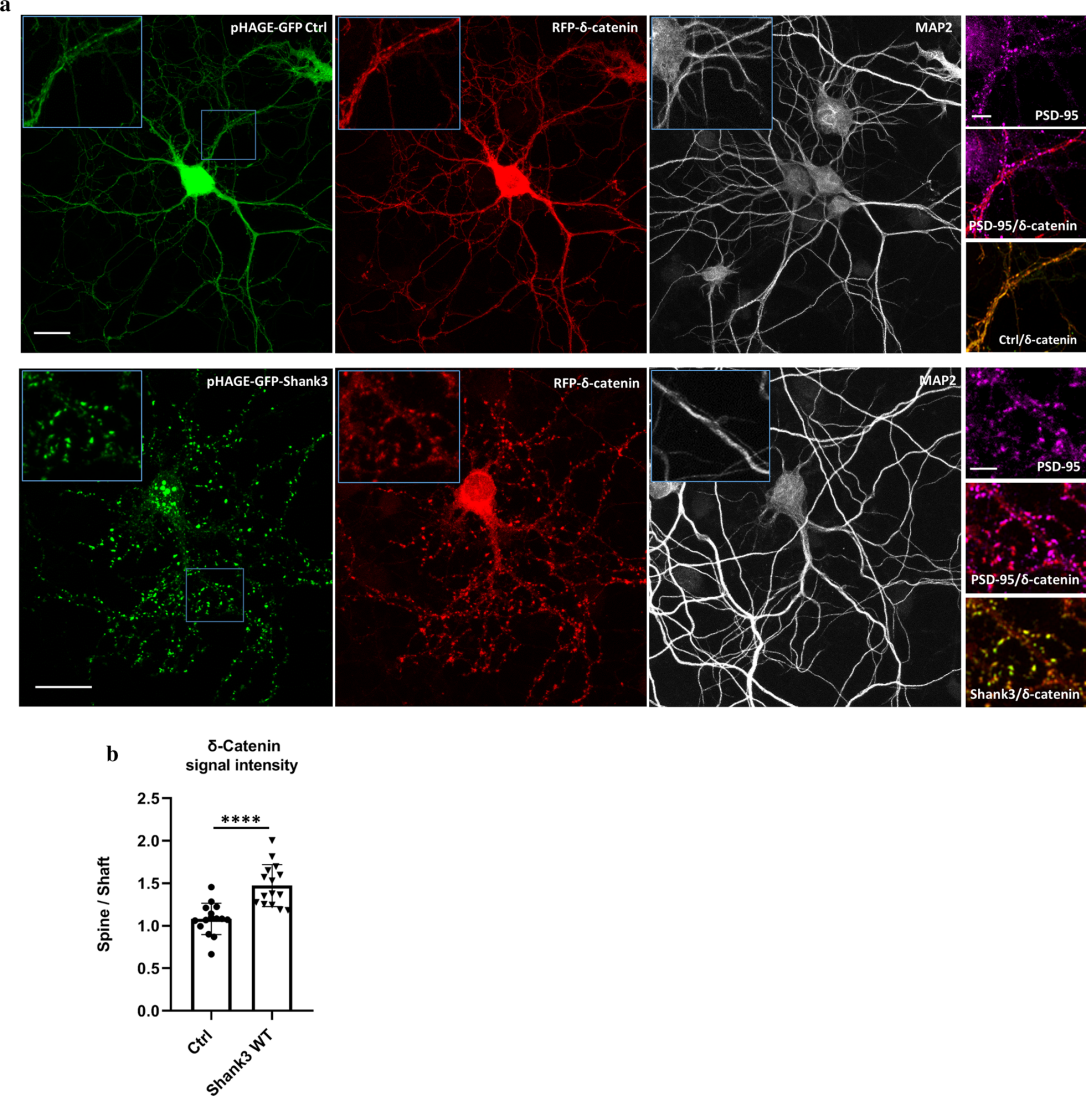
Effect of Shank3 on localization of δ-catenin in neurons. **a** Primary rat hippocampal neurons were transfected (DIV7) with RFP-δ-catenin together with either an empty pHAGE-GFP construct as control or pHAGE-GFP-Shank3 construct. The neurons were fixed (DIV14) and stained for MAP2 as dendritic marker and PSD-95 as synaptic marker (scale bar 20 µm). In the control condition δ-catenin shows a diffused signal throughout transfected neurons, whereas coexpression of Shank3 results in recruiting more δ-catenin to postsynaptic sites with a distinct punctate pattern along dendrites. Boxed areas are magnified for GFP, RFP and PSD-95 signals (scale bar 5 μm). **b** Using ImageJ, targeting of overexpressed δ-catenin to the postsynaptic sites was quantified as ratio of fluorescence signal intensity in dendritic spines (positive for PSD-95) versus the signal intensity in adjacent dendritic shafts. 150 spines of 15 neurons obtained from three independent experiments per each condition were analysed. The results of quantitative analyses show that Shank3 significantly increases the level of δ-catenin in the postsynaptic sites (*n* = 15, unpaired *T* test, ****p < 0.0001, mean ± SD)

To further investigate the role of Shank3 in the postsynaptic targeting of δ-catenin, we analysed the localization of δ-catenin in cultured hippocampal neurons upon expression of Shank3 using confocal microscopy. Unfortunately, we found the commercially available δ-catenin antibodies not suitable for immunocytochemical staining of the endogenous δ-catenin protein, as specific staining could not be validated upon shRNA knockdown of the *Ctnnd2* mRNA. Therefore, we relied on localization of δ-catenin expressed as a fusion to a fluorescent protein, similar to previous studies [31]. We expressed RFP-tagged δ-catenin using the neuron-specific synapsin promoter, to avoid extensive overexpression. In these experiments, RFP-δ-catenin was diffusely distributed throughout the neuron when expressed alone or together with GFP control (expressed from the empty pHAGE-GFP vector). Coexpression with pHAGE-GFP-tagged Shank3 dramatically changed this, as δ-catenin was now found to be highly colocalized with Shank3 in a typical postsynaptic pattern (Fig. 6a). Comparing the ratio of δ-catenin fluorescence signal intensity in the dendritic spines (positive for PSD-95 as postsynaptic marker) versus the intensity of signals in adjacent dendritic shafts shows that Shank3 significantly increases the level of δ-catenin at postsynaptic sites (Fig. 6b). This is in agreement with the findings using the isolated PSD fractions of WT and *Shank3* KO mice.

**Fig. 7:**
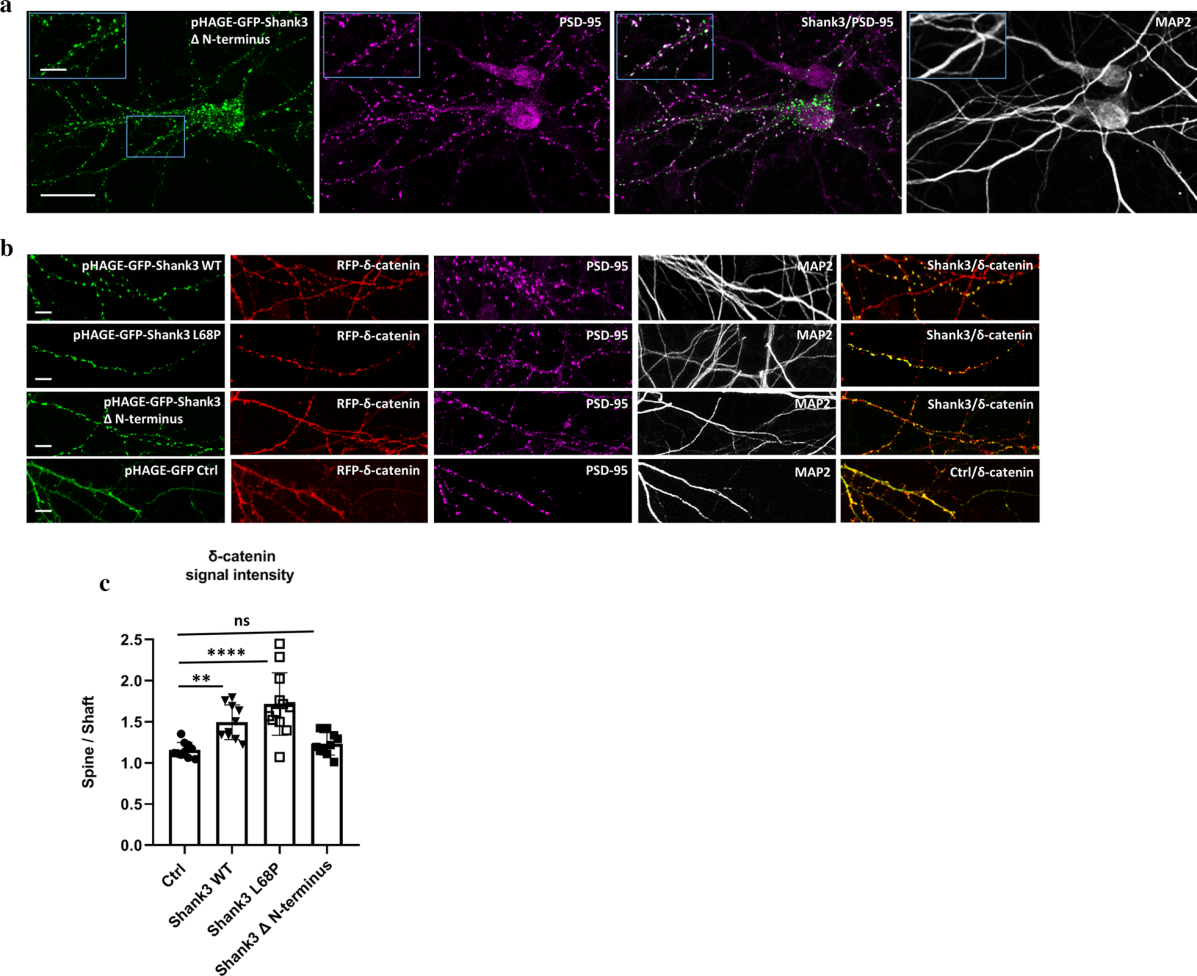
Contribution of the Shank3 N-terminus to the postsynaptic targeting of δ-catenin. **a** A new Shank3 construct lacking the N-terminal SPN-Ank domain was used for transfection of primary rat hippocampal neurons. The transfected neurons were fixed and stained for MAP2 and PSD-95. The green signal of Shank3 shows a high colocalization rate with PSD-95 indicating that the synaptic localization of Shank3 is not dependent on N-terminal domain (scale bar for overview images: 20 µm and for magnified images: 5 μm). **b** RFP-δ-catenin was coexpressed with either an empty pHAGE-GFP construct as a control or with different Shank3 (WT, L68P or ΔN-terminal) constructs. The neurons were fixed and stained for MAP2 as dendritic marker and PSD-95 as synaptic marker (scale bar 5 µm). **c** Quantitative analysis of δ-catenin signal intensity in 100 spines of 10 neurons obtained from two independent transfection, showed L68P variant of Shank3 increases the δ-catenin level in dendritic spines compared to Shank3 WT, whereas ΔN-terminal Shank3 is unable of changing localization of δ-catenin in neurons and shows a similar effect as pHAGE-GFP control construct. The results show that Shank3 N-terminus is responsible for recruiting δ-catenin to the postsynaptic sites (*n* = 10, one-way ANOVA with Dunnett’s Test, **p < 0.01, ****p < 0.0001, mean ± SD)

To clarify whether the observed increased targeting of δ-catenin to postsynaptic sites upon expression of Shank3 is mediated via a direct interaction with the Shank3 N-terminus, or is a result of other indirect interactions in the PSD, we designed a *Shank3* construct lacking coding sequence for the N-terminal SPN and Ank domains. The shortened Shank3 protein expressed from this construct was efficiently targeted to postsynaptic sites as it was found to be highly colocalized with PSD-95 (Fig. 7a). Interestingly, expression of this Shank3 construct did not show any significant effect on the targeting of overexpressed δ-catenin to dendritic spines in primary hippocampal neurons, as the obtained spine/shaft signal ratio was very similar to the control condition where we coexpressed the pHAGE-GFP empty vector. Coexpression of the ASD-associated Shank3 variant L68P with δ-catenin caused a strong increase in the postsynaptic localization of δ-catenin; this appeared somewhat stronger than the effect caused by Shank3 WT; however, the difference between mutant and WT did not become statistically significant (Fig. 7b, c).

## Discussion

The role of the N-terminal SPN and Ank domains for the synaptic function of Shank3 is unclear. So far, HCN1, Fodrin and Sharpin have been reported as interaction partners of the Shank1 and Shank3 Ank domains [13–15]. Whereas the presence of Sharpin in the postsynaptic density is barely noticeable, both α-fodrin and HCN1 are present in the PSD and seem to be good candidates. However, binding of α-fodrin to the Ank domain is inhibited by the presence of the SPN domain, and the relevance of the SPN domain for HCN1 binding is unclear. Importantly, as there is no similarity between α-fodrin and HCN1, it is unclear which motifs mediate binding to the Ank domain. Therefore, we aimed to identify further interaction partners for the Shank3 N-terminus, trying to take into account the presence of the SPN domain which is tightly associated with the Ank repeats.

Using a His_6_/SUMO fusion protein that contains the N-terminus of Shank3 as an affinity matrix to purify possible interacting proteins from the postsynaptic density led us to the catenin family of proteins. We identified several family members including α-, β- and δ-catenin to be enriched upon precipitation with the Shank3 N-terminus. As catenins interact with each other directly and indirectly (through cadherins), an obvious possibility was that some catenins were bound to the Shank3 N-terminus indirectly. When tested individually upon coexpression with full-length Shank3 in 293T cells, we observed that δ-catenin highly colocalizes with Shank3. Importantly, δ-catenin led to a partial translocation of Shank3 to the plasma membrane where δ-catenin is localized due to its palmitoylation [31]. No colocalization was observed with α-catenin. In addition, we have shown previously that β-catenin colocalizes with Shank3 in the nucleus upon coexpression [23].

In agreement with these microscopic data, verification of the mass spectrometric results by coexpression in cultured cells, followed by co-immunoprecipitation, showed that both β- and δ-catenin (but not α-catenin) can bind to Shank3, but only δ-catenin interacts with the N-terminal domain of Shank3. It is likely that in the PSD, which we used as starting material for purification, all these proteins are in complex together. Therefore interaction of δ-catenin with the isolated N-terminal fragment of Shank3 may have resulted in appearance of the other catenins in the mass spectrometric results. Importantly, previous work in mice expressing a GFP-tagged form of Shank3 has shown that Shank3 is in a complex with δ-catenin in vivo in the brain [25]

Performing coimmunoprecipitation assays using different truncated fragments of δ-catenin showed that the Armadillo repeat region of this protein makes contact with Shank3, as the deletion of Armadillo repeats almost abolished the interaction with Shank3. On the other hand, the SPN domain was dispensable for interaction with δ-catenin, as in our pulldown experiments with SUMO-tagged fusion proteins, the Ank domain alone was sufficient to bind to δ-catenin. In this respect, δ-catenin differs from α-fodrin; α-fodrin binding to the Ank repeats of Shank3 is inhibited by the presence of the SPN domain which binds to the Ank repeats in an intramolecular interaction [16]. Surprisingly, in this set of experiments we did not observe any interaction of the Shank3 N-terminus with HCN1, regardless whether the SPN domain was present or not. Currently we can speculate whether a larger fragment of the Shank3 N-terminus is required to elicit interaction with HNC1; however, attempts to coimmunoprecipitate HCN1 with full-length Shank3 from transfected cells failed as we observed only no, or non-specific interaction.

Investigating the effect of seven ASD-associated missense mutations in the Shank3 N-terminus on the interaction between Shank3 and δ-catenin revealed that only the L68P mutation in the SPN domain significantly improves the binding of δ-catenin to the Ank domain of Shank3. However, the increase was rather small when compared to the large increase induced by this mutation on α-fodrin binding [16]. The L68P mutation inactivates the SPN domain, possibly through local unfolding [12]. Taken together, our binding analyses indicate that δ-catenin binds to a surface on the Ank domain of Shank3 that is only partially overlapping with the binding site for α-fodrin, and is therefore less sensitive to the presence of the SPN domain [16].

In 2016 Wang et al. reported δ-catenin as a neuronal substrate for TNIK, a serine/threonine kinase highly expressed in the brain and enriched in the postsynaptic density. Identification of multiple TNIK phosphorylation sites of δ-catenin suggests phosphorylation by TNIK is particularly important in synaptic adhesion, and synaptic plasticity [30]. To investigate whether δ-catenin phosphorylation via TNIK affects the binding to Shank3, we coexpressed the WT or a kinase dead TNIK constructs with Shank3 and δ-catenin. In addition, we also mutated one of the phosphorylation sites (T1064) to alanine or to glutamate. The results of coimmunoprecipitation showed that the presence of an active TNIK or mutation of this residue does not affect the interaction between Shank3 and δ-catenin. However, these results do not rule out the possibility that other posttranslational modifications affect the Shank3/δ-catenin interaction. The phosphosite database (https://www.phosphosite.org) lists more than 40 phosphorylation events in δ-catenin, several of which affects residues in the Armadillo repeat section. In addition, S-nitrosylation of proteins has been shown to differentially affect protein interactions in *Shank3* KO vs WT mice and should be considered as a factor here [33].

To examine the effect of the Shank3 N-terminus on the postsynaptic localization of δ-catenin, we compared the δ-catenin content of an isolated postsynaptic fraction of *Shank3* KO mice to a postsynaptic fraction isolated from WT mice. Interestingly, we found that the level of postsynaptic δ-catenin in *Shank3* KO mice lacking the largest isoform of Shank3 containing the N-terminal domain is significantly reduced; however the level of β-catenin was not affected in the postsynaptic density of the same animals. By comparing the P2 membrane fractions of *Shank3* KO and WT mice we observed no significant differences between WT and KO animals in their δ-catenin content. These result indicate that whereas total δ-catenin protein levels are not changed in the *Shank3* KO mice, a lack of the Shank3 N-terminus results in loss of postsynaptic δ-catenin. Thus, Shank3 is crucially involved in the postsynaptic localization and stabilization of δ-catenin through the interaction mediated by the Shank3 N-terminus. Interestingly, we have observed here that Shank1 is also able to interact with δ-catenin; the remaining Shank1 may be responsible for maintaining residual δ-catenin in the postsynaptic density of *Shank3* KO mice.

These results are fully supported by our microscopic analysis of δ-catenin localization in cultured hippocampal neurons. It is known that, under basal conditions, δ-catenin is highly localized to dendritic shafts; however, neuronal activity promotes DHHC5-mediated palmitoylation of δ-catenin and subsequently its trafficking to the postsynaptic density [31]. In agreement with this observation, expression of a RFP-tagged δ-catenin in primary cultured neurons showed a diffuse pattern of localization throughout the neuron with a similar signal intensity in dendritic spines and dendritic shafts (spine/shaft ratio ~ 1). Coexpression with Shank3 significantly increased the signal intensity of δ-catenin in dendritic spines compare to their adjucent dendritic shafts (spine/shaft ratio ~ 1.5). Further, we showed that an overexpressed Shank3 variant lacking the N-terminal SPN and Ank domains fails to recruit δ-catenin to postsynaptic sites. As a result δ-catenin remains diffusely distributed throughout the neuronal dendrites and shows a similar pattern as when coexpressed with GFP alone. However, the overexpression of the ASD-associated L68P variant improved the signal intensity of δ-catenin slightly when compared to WT. Altogether our data indicate that interaction with the Shank3 N-terminus provides an additional mechanism to target δ-catenin to the PSD. As we observe that interaction with Shank3 is not dependent on palmitoylation of δ-catenin at Cys960/961, we assume that interaction with Shank proteins is required for a basal level of postsynaptic δ-catenin, whereas the activity dependent palmitoylation of δ-catenin may increase these levels in periods of synaptic plasticity.

Interestingly, δ-catenin dominates Shank3 localization in 293T cells, as it targets Shank3 to the plasma membrane. In neurons, this situation is reversed, as Shank3 targets δ-catenin to dendritic spines. This may be due to the fact that in 293T cells, Shank3 lacks its complement of postsynaptic partners such as Homer or GKAP/SAPAP proteins. Thus it can be affected by δ-catenin, as its only partner which may affect localization. In neurons, δ-catenin needs palmitoylation to be at the synapse [31], but apparently this is not sufficient as we observe here the need for interaction with Shank proteins.

One might ask why the levels of δ-catenin need to be controlled in two different ways, and which function δ-catenin has at postsynaptic sites. δ-catenin and p120ctn both belong to the p120ctn family of catenins which decrease endocytosis of surface cadherins. As a result, loss of the members of this family reduces cadherin stability at postsynaptic sites [34, 35]. Here, we asked whether the postsynaptic level of N-cadherin is affected by the reduced level of δ-catenin in the postsynaptic density of *Shank3* KO mice. Comparing the PSD fraction of *Shank3* KO and WT mice showed a slight but not significant reduction in the postsynaptic levels of N-cadherin in the KO mice. This result may be explained by a likely compensatory effect of the other catenins such as β-catenin and p120ctn present in the postsynaptic density of *Shank3* αβ − / − mice that prevent a drastic loss of N-cadherin in synapses.

In addition, it has been shown that δ-Catenin promotes surface expression of AMPA receptors and results in enhanced AMPA receptor-mediated synaptic currents [36]. Also, loss of δ-catenin is associated with a decrease in overall excitatory synapse density, as well as active synapses that express the GluA subunit of the AMPA receptors [32, 37]. Although we have not investigated the surface expression of AMPA receptors in this study, our findings presented here might indicate a novel mechanism in which Shank3 controls synaptic activities through regulating the postsynaptic level of δ-catenin. This regulatory effect of Shank3 is strongly mediated by the N-terminal SPN-Ank domain, where mutating or eliminating this domain results in drastic changes in the level of δ-catenin in the postsynaptic sites.

The human δ-catenin gene (*CTNND2*) is located on chromosome 5p15.2 where a deletion causes the *cri-du-chat* syndrome (CDCS), a syndrome with severe cognitive and language impairments, motor delays, and behavioural problems [38]. *CTNND2* has been implicated as an autism candidate gene, as several deletions and unbalanced translocations have been reported in individuals with autism and other neurodevelopmental disorders [9]. Though not many cases have been observed, *CTNND2* is somewhat similar to *SHANK3* as alterations in both genes are associated with multiple neurological disorders. Our data indicate that, through their interaction at postsynaptic sites, Shank3 and δ-Catenin contribute to a synaptic signalling pathway which is disrupted in ASD and other neurodevelopmental disorders. An interesting experiment here would be to cross the *Shank3* and *Ctnnd2* KO mice, to observe whether their phenotypes with respect to neuron morphology as well as behaviour are additive. It would also be interesting to see whether Shank3 expression can rescue deficits in synaptic targeting of δ-catenin under conditions of defective palmitoylation.

## Conclusions

We show that δ-catenin is an interaction partner of the N-terminal Ankyrin repeats of Shank3. This interaction is necessary for postsynaptic targeting of δ-catenin. Our data indicate that, through their interaction at postsynaptic sites, Shank3 and δ-catenin contribute to a synaptic signalling pathway which is disrupted in ASD and other neurodevelopmental disorders.

### Limitations

We see the usual limitations applying to studies in model organisms such as mice (used here in Fig. 5), namely the limited transferability of the data to the human situation. Also work in primary cultured neurons, as shown here in Figs. 6 and 7, may not reproduce faithfully the situation in human brain neurons. Work in primary cultured neurons was also hampered by lack of a specific antibody for endogenous δ-catenin; we tried to overcome this by expression using a neuronal-specific promoter. Finally, we have used cDNA constructs from rodents for our analysis (partly because we used expression in rodent model systems). Again, these sequences may not completely behave in the same way as the human sequence. However, there is a very high level of sequence identity e.g. between rodent and human Shank3, in particular in the Ank of Shank3 domain which is the focus of analysis here.

## Supplementary information

**Additional file 1**. Supplementary information.

## Data Availability

All data generated or analysed during this study are included in this published article [and its supplementary information files].

## Abbreviations

Ank: Ankyrin repeats
ANOVA: Analysis of variance
Arm: Armadillo
ASD: Autism spectrum disorder
CMV: Cytomegalovirus
Ctrl: Control
DAPI: 4′,6-Diamidino-2-phenylindole
DIV: Days in vitro
DOC: Desoxycholate
EDTA: Ethylenediaminetetraacetic acid
EGFP: Enhanced green fluorescent protein
HCN1: Hyperpolarization activated cyclic nucleotide gated channel 1
HEK: Human embryonic kidney
His: Histidine
HRP: Horseradish peroxidase
IP: Immunoprecipitation
KM: Kinase mutant
KO: Knock out
LC-MS: Liquid chromatography–mass spectrometry
MAP2: Microtubule-associated protein 2
mRFP: Monomeric red fluorescent protein
MS: Mass spectrometry
NCAD: N-cadherin
PAGE: Polyacrylamide gel electrophoresis
PBS: Phosphate-buffered saline
PCR: Polymerase chain reaction
PDZ: PSD-95/DLG/ZO1
PSD: Post-synaptic density
*PSD-95*: Postsynaptic density protein 95
RIPA: Radioimmunoprecipitation assay
SAM: Sterile alpha motif
SD: Standard deviation
SDS: Sodium dodecyl sulfate
SH3: Src Homology 3
Shank: SH3 and multiple ankyrin repeats
shRNA: Short hairpin RNA
SPN: Shank3/ProSAP N-terminal
SUMO: Small Ubiquitin-like Modifier
SynGAP: Synaptic Ras GTPase-activating protein 1
TBS-T: Tris-buffered saline-Tween 20
TNIK: TRAF2 and NCK-interacting protein kinase
WB: Western blot
WT: Wild type
