# Supplementary material for: Targeting of δ-catenin to postsynaptic sites through interaction with the Shank3 N-terminus
Source: Mol Autism. 2020 Oct 28;11:85. doi: 10.1186/s13229-020-00385-8 (PMC7592556; doi:10.1186/s13229-020-00385-8)
Supplement: Supplementary file 1 — Additional file 1. Supplementary information. [file 13229_2020_385_MOESM1_ESM.pdf]

Supplementary Figures for

Targeting of δ-catenin to postsynaptic sites through an interaction with the Shank3 N-terminus suggests functional cooperation of two autism risk factors

Fatemeh Hassani Nia[1], Daniel Woike[1], Victoria Martens[1], Malte Klüssendorf[1,2], Hans-Hinrich Hönck[1], Sönke Harder[3] and Hans-Jürgen Kreienkamp[1]*

[1]Institut für Humangenetik, Universitätsklinikum Hamburg-Eppendorf, Hamburg, Germany

[2]Institut für Osteologie und Biomechanik, Zellbiologie seltener Erkrankungen, Universitätsklinikum Hamburg-Eppendorf, Hamburg, Germany

[3]Massenspektrometrische Proteomanalytik; Institut für klinische Chemie und Laboratoriumsmedizin; Universitätsklinikum Hamburg-Eppendorf, Hamburg, Germany

*To whom correspondence should be addressed at

Institut für Humangenetik
Universitätsklinikum Hamburg-Eppendorf
Martinistrasse 52
20246 Hamburg, Germany
e-mail: Kreienkamp@uke.de

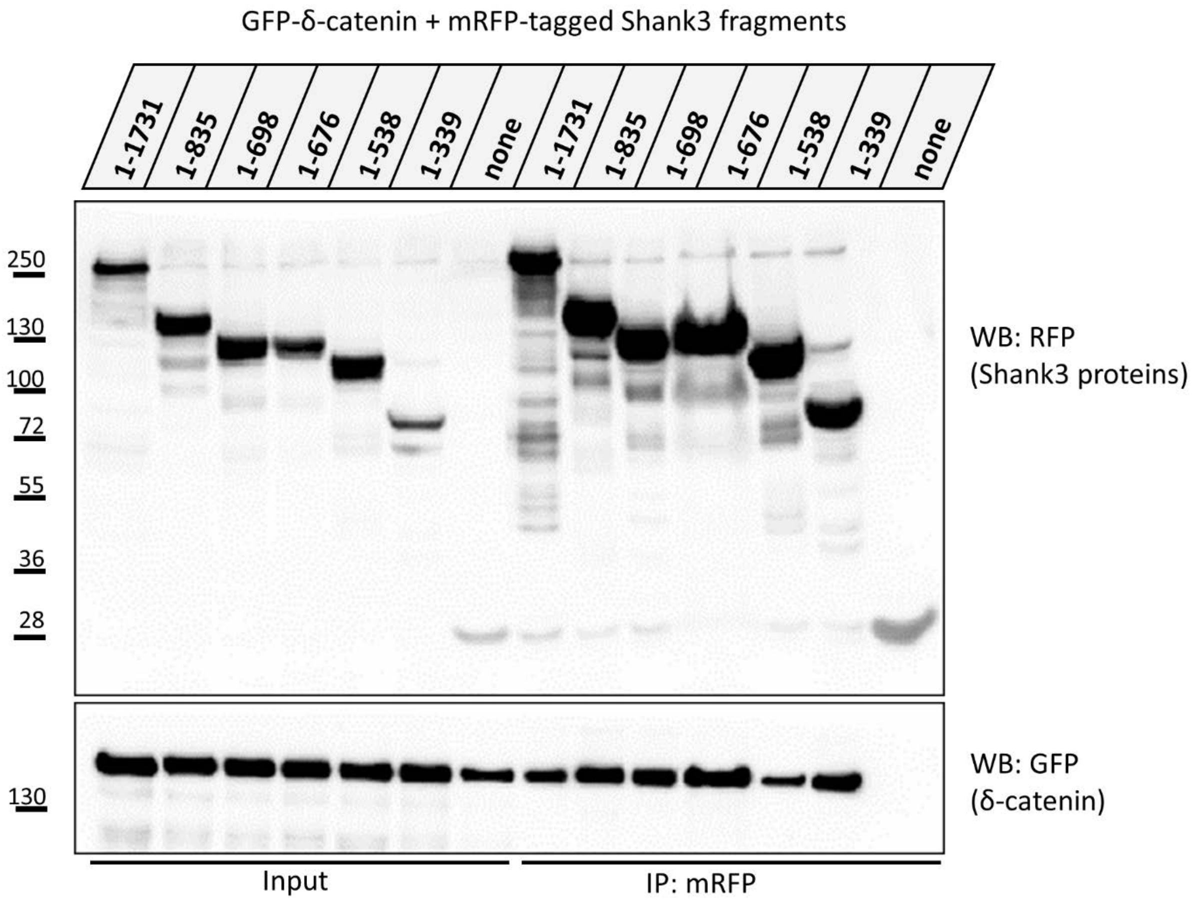

Supplementary figure 1. Mapping the binding sites for δ-catenin on Shank3. 293T cells coexpressing mRFP-Shank3 fragments with GFP-tagged δ-catenin were lysed. The results of immunoprecipitation with RFP-trap show that δ-catenin binds to all fragments of Shank3 tested, including the shortest truncated Shank3 protein which contains the N-terminal SPN-Ank domains.

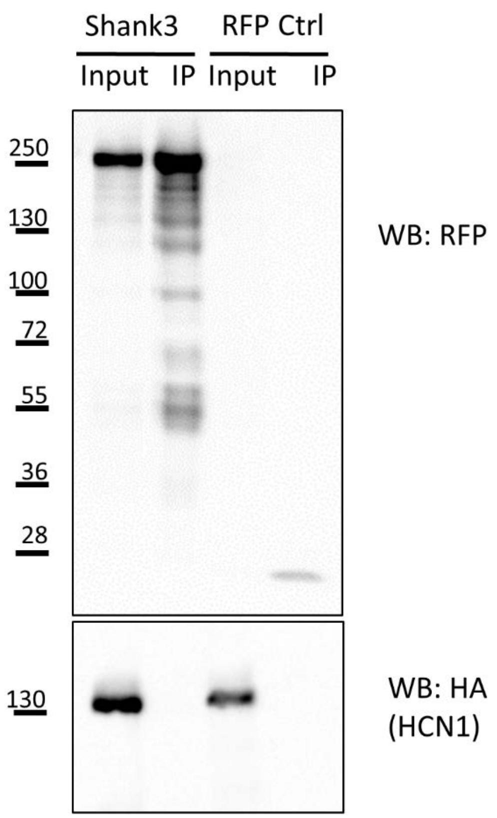

Supplementary figure 2. No interaction betweenShank3 and HCN1. 293T cells coexpressing HA-tagged HCN1 together with either mRFP-Shank3 or an mRFP construct as a control were lysed and subjected to immunoprecipitation using RFP-trap. Western blot result shows that similar to the control condition with RFP, HCN1 does not coprecipitate with Shank3.

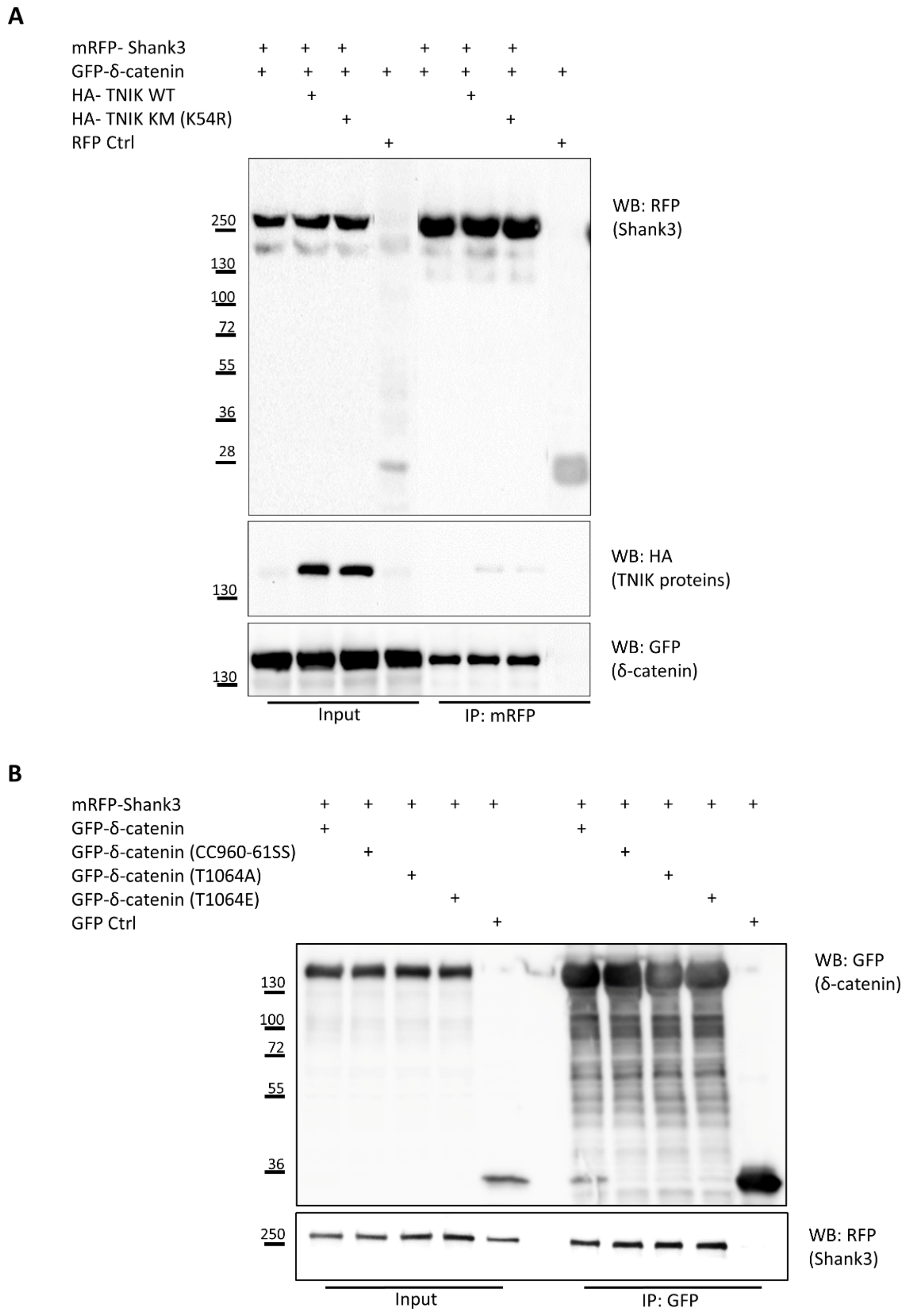

Supplementary figure 3. No effect of posttranslational modifications of δ-catenin on interaction with Shank3. A. 293T cells overexpressing GFP-δ-catenin and mRFP-Shank3 without TNIK or in the presence of either HA-TNIK WT or the TNIK "kinase dead" mutant K54R were lysed. Immunoprecipitation was performed using RFP-trap beads. Comparing the amount of coprecipitated GFP-δ-catenin in all three conditions showed that the presence of TNIK and most likely the phosphorylation of GFP-δ-catenin via TNIK does not affect the interaction of GFP- δ-catenin with Shank3. **B.** 293T cells were cotransfected with mRFP-Shank3 and GFP-tagged mutated variants of δ-catenin, as indicated. A coimmunoprecipitation was performed using GFP-trap. The results showed that mutation in neither the phosphorylation site (T1064E, T1064A) nor palmitoylation site (CC960-61SS) of δ-catenin affect the binding to Shank3.

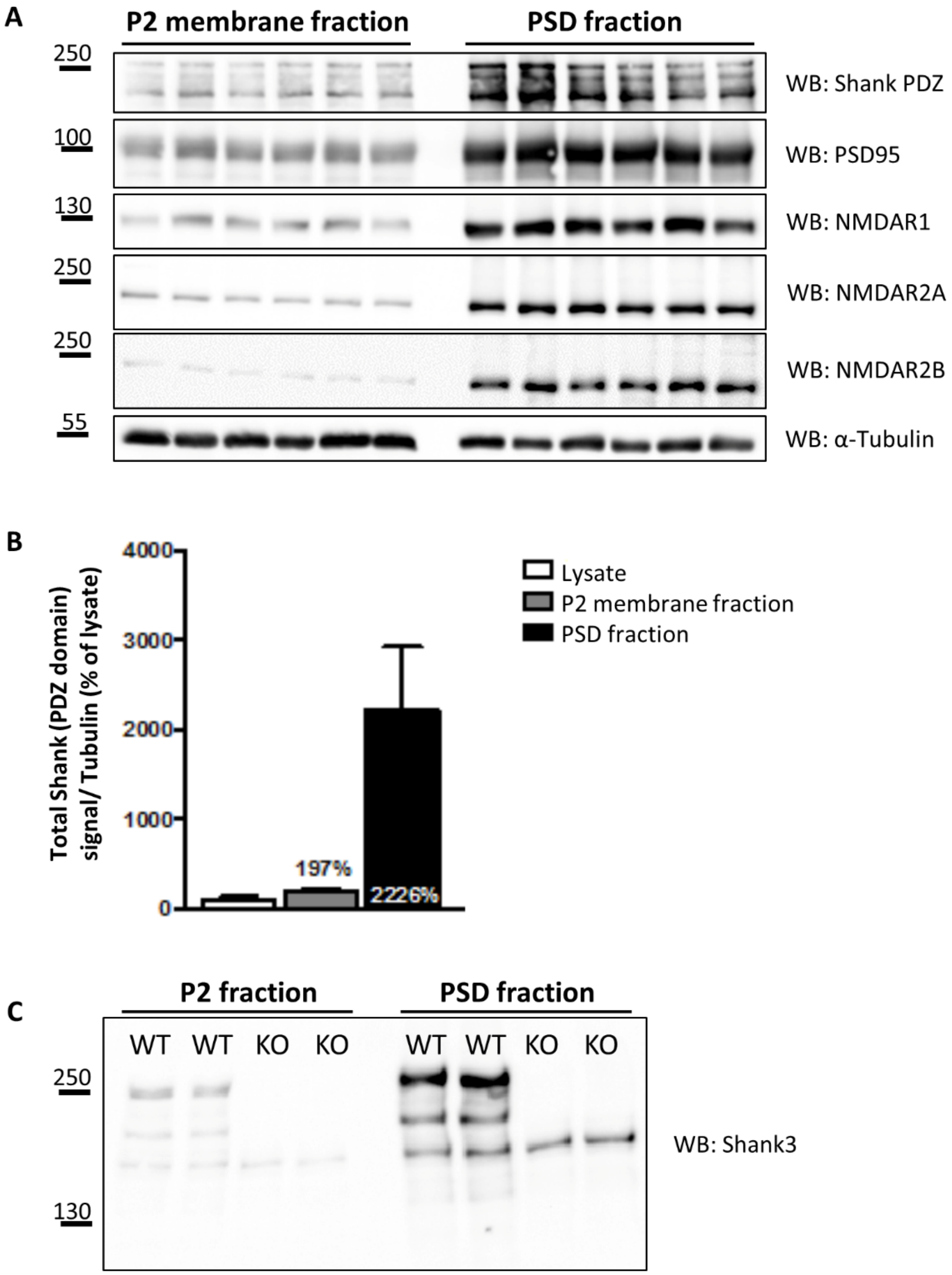

Supplementary figure 4. Enrichment of postsynaptic proteins in the PSD fraction. A. P2 membrane fractions and the PSD fraction were prepared from the brains of six mice. Equal amounts of protein were analysed by Western Blotting using the antibodies indicated. Shank PDZ recognizes all three Shank variants, Shank1-3. **B.** Enrichment of the Shank PDZ signal (all bands) is shown from brain homogenates via the P2 fraction to the PSD fraction. Intensity of the Shank signal is normalized to the Tubulin signal, which is consistently weaker in PSD fractions compared to P2. **C.** Enrichment of the Shank3 protein from P2 membrane to PSD fraction, shown for WT and *Shank3* KO mice. Note that the two largest Shank3 bands are missing, in agreement with loss of isoforms containing SPN, Ank and SH3 domains.
